# Conducting polymer-coated MIL-101/S composite with scale-like shell structure for improving Li–S batteries

**DOI:** 10.1039/c7ra12800b

**Published:** 2018-01-26

**Authors:** Wen-Wu. Jin, He-Jun. Li, Ji-Zhao. Zou, Shao-Zhong. Zeng, Qing-Duan. Li, Guo-Zhong. Xu, Hong-Chao. Sheng, Bei-Bei. Wang, Yun-Hui. Si, Liang. Yu, Xie-Rong. Zeng

**Affiliations:** State Key Laboratory of Solidification Processing, Carbon/Carbon Composites Research Center, Northwestern Polytechnical University Xi'an 710072 China; Shenzhen Key Laboratory of Special Functional Materials & Shenzhen Engineering Laboratory for Advance Technology of Ceramics, College of Materials Science and Engineering, Shenzhen University Shenzhen 518060 PR China zengxier@szu.edu.cn zoujizhao@szu.edu.cn; School of Materials Science and Engineering, Jiangsu University of Science and Technology Zhenjiang 212003 China

## Abstract

Lithium–sulfur batteries are regarded as a promising energy storage system. However, they are plagued by rapid capacity decay, low coulombic efficiency, a severe shuttle effect and low sulfur loading in cathodes. To address these problems, effective carriers are highly demanded to encapsulate sulfur in order to extend the cycle life. Herein, we introduced a doped-PEDOT:PSS-coated MIL-101/S multi-core–shell structured composite. The unique structure of MIL-101, large specific area and conductive shell ensure high dispersion of sulfur in the composite and minimize the loss of polysulfides to the electrolyte. The doped-PEDOT:PSS-coated sulfur electrodes exhibited an increase in initial capacity and an improvement in rate characteristics. After 192 cycles at the current density of 0.1C, a doped-PEDOT:PSS-coated MIL-101/S electrode maintained a capacity of 606.62 mA h g^−1^, while the MIL-101/S@PEDOT:PSS electrode delivered a capacity of 456.69 mA h g^−1^. The EIS measurement revealed that the surface modification with the conducting polymer provided a lower resistance to the sulfur electrode, which resulted in better electrochemical behaviors in Li–S battery applications. Test results indicate that the MIL-101/S@doped-PEDOT:PSS is a promising host material for the sulfur cathode in the lithium–sulfur battery applications.

## Abstract 2

Lithium–sulfur batteries are regarded as a promising energy storage system.

## Graphical abstract

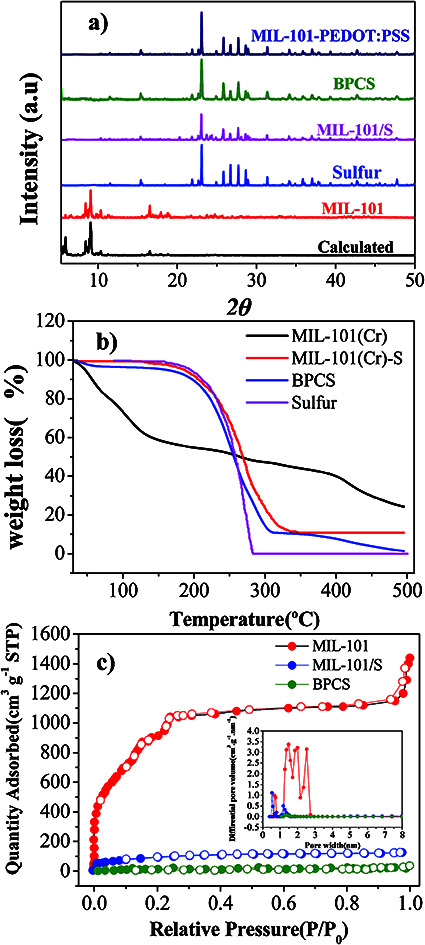

## Introduction

Lithium–sulfur batteries have an unparalleled theoretical gravimetric capacity (1675 mA h g^−1^). As a result, they are strong contenders for replacing lithium-ion batteries as a next-generation energy storage technology. However, Li–S batteries have yet to be commercialized due to unsolved issues that remain despite the constant effort of research over many years. These unsolved issues include: (1) the poor conductivity of both elemental sulfur (5 × 10^−30^ S cm^−1^ at 25 °C) and its discharge product Li_2_S, which obstructs the electron transfer of the charge–discharge reactions;^1,2^ (2) the dissolution of polysulfides and the resulting shuttling effect during the charge–discharge process, which leads to the loss of active material, low coulombic efficiency and high interfacial impedance at lithium anode;^1^ (3) the large volume expansion of S (80%) upon lithiation.^2^

In order to overcome those problems mentioned above, two strategies were proposed. First of all, porous conductive carbon materials, such as various hollow (nanospheres^3^), flexible (carbon paper,^4^ grapheme,^5^ nanoribbons^6^) or carbon tunnel materials (carbon nanotubes,^7^ carbon nanofibers),^8^ have been employed as matrix materials, which not only reduce the charge transfer resistance of composite but also encapsulate sulfur and limit the shuttle effect originating from soluble lithium polysulfides. Besides major efforts have been centered on the development of novel core–shell structure composites of sulfur and conductive polymers,^9–16^ metallic oxides^17^ or graphite or reduced grapheme oxide,^18,19^*etc.* In addition to the methods mentioned above, the researchers were surprised to discover that combining the methods mentioned above to produce a dual sulfur-retaining tactic would open new opportunities to rationally develop cathode materials with superior properties.^10,14^

Yi Cui *et al.*^16^ studied the effect of a coating of conducting polymer on a sulfur/carbon, using an additional heat-treatment process to obtain carbon/sulfur materials before coating with poly(3,4-ethylenedioxythiophene)–polystyrene sulfonic acid (PEDOT:PSS). Although the previous reports mentioned above addressed the improved electrochemical performances of sulfur electrodes, the cathodes have a “low sulfur content” of only about 50 wt% and a “low sulfur loading” of less than 2 mg cm^−2^. In fact, the issue of “two lows” significantly reduces the overall energy density per gram of cathode, although a very high specific capacity of sulfur can be obtained^20^ or in some cases, despite the sulfur content of the material being high, the sulfur loading of the electrode is still low, which also leads to a low energy density of the cathode^21^ Cathodes of high energy density are essential for high energy density batteries. It is necessary to improve the utilization of cathode materials and the sulfur loadings simultaneously.

The questions mentioned above have put forward higher requirements for the matrix material and the shell materials, which should have an extra-large surface area and a better electrical conductivity, respectively. Thanks to the emergence of metal organic frameworks (MOF), which open a new route to solve the problem of porous matrix material. Metal–organic frameworks (MOFs) are periodic, hybrid, atomically well-defined porous materials,^22^ which offer many intriguing properties, including ultrahigh porosity, low density, super-large specific surface area, adjustable pore size and topology diversity (0-dimension,^23^ one-dimensional,^24,25^ two-dimension,^26–29^ three-dimension).^30^

Besides, the conductivity and the wettability of shell material can be further modified by doping with a suitable dopant.^31–33^ It is well known that PEDOT:PSS aqueous dispersion is made up of a certain concentration of PSS in PEDOT. However, the insulating PSS that contains sulfonic acid SO_3_H groups may bring detrimental effects such as low conductivity and lifetime issues.^34^ Dimethyl sulfoxide (DMSO) and Triton X-100 are commonly used as co-solvents to modify the morphology and nanostructure of PEDOT:PSS, and the conductivity could be significantly improved.^31–33^

Herein, MIL-101(Cr) was used in this study as a sulfur host in Li–S batteries. MIL-101 (ref. 35) is selected because it possesses a large surface area (5000 m^2^ g^−1^) and pore volume (>1.6 cm^3^ g^−1^). The unique pore structure and high surface area would favor the high dispersion of sulfur into the pores with strong interactions. In addition, a biomolecule-doped PEDOT:PSS was used to coat the MIL-101(Cr)/S composite in order to build a conductive bridge for electron transfer and simultaneously a physical barrier to curb the dissolution of polysulfides into the liquid electrolyte. The strong binding affinity of PEDOT with Li_*x*_S (0 < *x* ≤ 2) can effectively reduce the polysulfide diffusion into the electrolyte and thus contribute to a more stable cycling performance.^36^

**Scheme 1:**
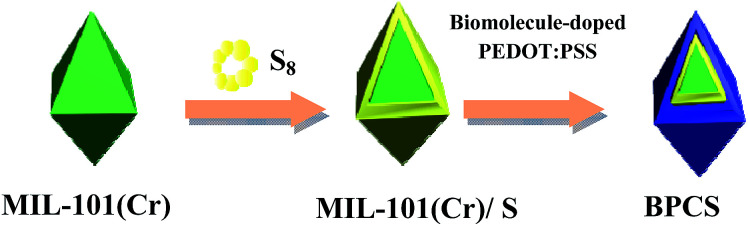
Illustration of the synthesis route for the BPCS multi-core–shell structured composite.

**Scheme 2:**
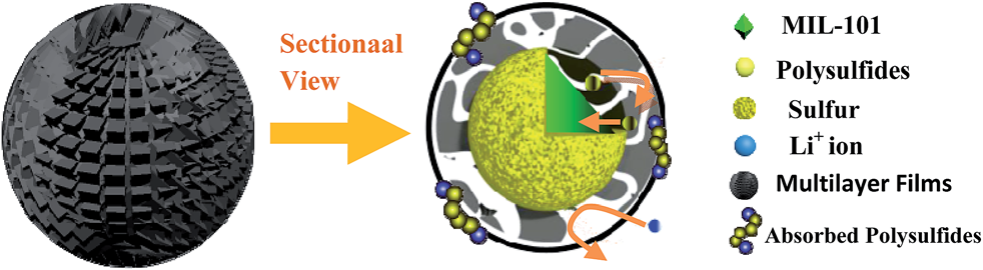
The schematic illustration of the limitation of lithium polysulfides in multilayer film structure.

As schematically illustrated in Scheme 2, the final product is denoted as biomolecule-doped PEDOT:PSS/Cr-MIL-101/sulfur (BPCS). Scheme 1 illustrates the schematics of a BPCS composite synthesis by a two-step approach. Unlike the complicated preparation process of matrix carbon material, the MIL-101 can be obtained *via* a facile one-step hydrothermal method,^35^ and the high yields of the products provide a guarantee for industrial production. Sulfur embedded in multi-core–shell structured composite exhibits high specific capacities and excellent cycling stabilities. At a current rate of 0.1C, the BPCS composite still delivers a discharge capacity of 606.62 mA h g^−1^ after 192 th cycles and the coulombic efficiency is about 99.1%, at the same time, the preparation process is simple and scalable. The prepared BPCS composite cathode possesses a much better capturing ability of the polysulfides and high electron conductivity. Thus, improved cycling stability and rate capability are achieved as shown subsequently.

## Results and discussion

### Characterization of MIL-101/S@PEDOT:PSS and BPCS

The morphologies of the as-prepared sulfur particles, S/MIL-101, MIL-101/S@PEDOT:PSS, and BPCS, were investigated by SEM. SEM (Fig. 2(a–d)) images acquired from the composite of MIL-101(Cr) and MIL-101(Cr)/S show that most of the MIL-101(Cr) particles were covered by sulfur uniformly. Moreover, the wrapped MIL-101(Cr)/S particles (Fig. 2(c and d)) preserve the original morphology and particle size of the MIL-101 without any agglomeration of sulfur on the particle surface. This indicates a high dispersion of sulfur into the porous framework matrix.

**Fig. 1:**
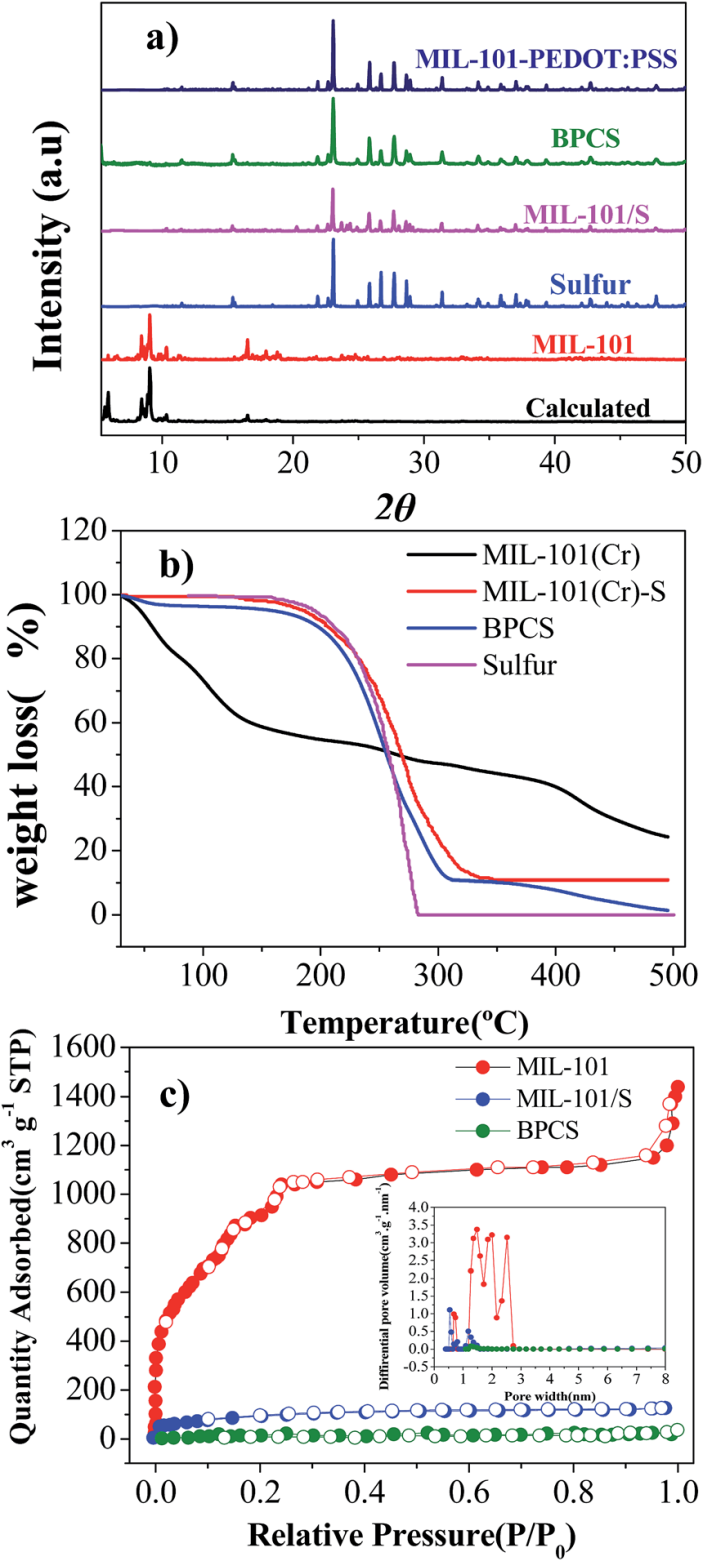
(a) X-ray diffraction patterns of calculated MIL-101(Cr), MIL-101(Cr), sulfur, MIL-101(Cr)/S, MIL-101/S@PEDOT:PSS, and BPCS composites; (b) TGA curves of MIL-101 (Cr), MIL-101(Cr)/S and BPCS composites; (c) nitrogen adsorption/desorption isotherms and PSD curves calculated from DFT theory for the MIL-101(Cr), MIL-101(Cr)/S and BPCS.

**Fig. 2:**
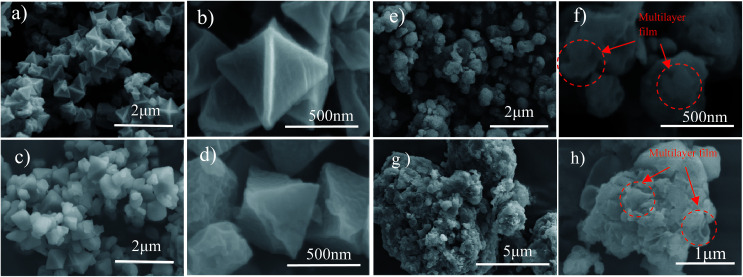
(a, b)MIL-101(Cr); (c, d)MIL-101(Cr)/sulfur composite; (e, f) BPCS; (g, h) MIL-101/S@PEDOT:PSS composite.

A repeated coating process was used to apply a layer on MIL-101(Cr)/S particles in this study, 5wt% dimethyl sulfoxide (DMSO) and 1wt% hexaethylene glycol monododecyl ether (Triton X-100) were added into the aqueous PEDOS:PSS dispersion (Clevios PH 1000) to improve the conductivity and adhesion of PEDOT:PSS on MIL-101(Cr)/S particles, therefore, the two composites had markedly different microstructures.

**Fig. 3:**
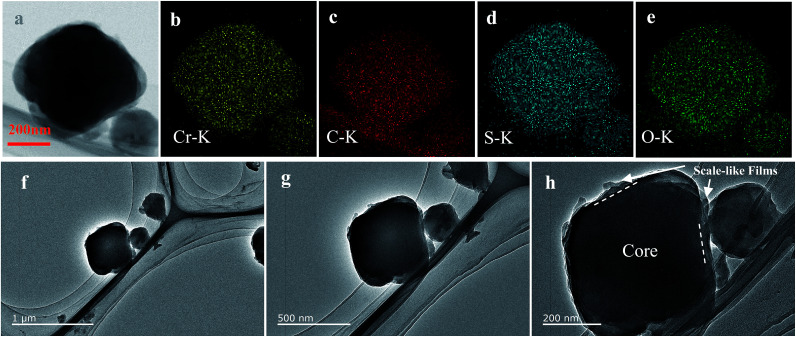
TEM image (a, f–h) of BPCS and the corresponding EDS elemental maps for chromium (b) carbon (c) sulfur (d) oxygen (e).

Agglomeration of PEDOT:PSS modified MIL-101(Cr)/S powder (Fig. 2(g and h)) is improved by doping 5 wt% DMSO and 1 wt% Triton X-100. The BPCS composite shows that most of the MIL-101(Cr)/S particles were covered by doped-PEDOT:PSS uniformly and tightly, and the MIL-101/S particles were very smooth and in intimate contact with the doped-PEDOT:PSS (Fig. 2(e and f)). At the same time, it was worth notice that each composite layer of BPCS was ultrathin and its thickness could be controlled by repeating the coating process. The particle size of BPCS increased very slightly (Fig. 2 and 3). Furthermore, it can also be seen from Fig. 2(f and h) and 3(h) that the ultra-thin film prepared by the cyclic process has a multilayer scale-like structure (Scheme 2). Although the structure of the shell was incomplete, the structure of multilayer film could minimize the exudation of internal material and ensure the penetration of electrolyte. If the pre-assembly wrapping layer was designed to be closely compact and tight so that the electrolyte would be blocked, the electrolyte would not infiltrate the C/S composite in the assembled cell. Therefore, the battery would exhibit poor performance. Alternatively, if the pre-assembly coated layer was imperfectly designed with pores and/or cracks that allow for the penetration of the electrolyte into the C/S composite, solvated polysulfides could also leak out of the wrapping layer *via* these defects, which leads to an improved but still diminishing capacity.^37^

To further confirm the distribution of elemental S in the composite, the HAADF-STEM image and the corresponding element mapping of chromium, sulfur, oxygen and carbon are shown in Fig. 3. Fig. 3(a) shows the doped-PEDOT:PSS coated bipyramidal shaped MIL-101/S particles. The elemental distribution maps on a single BPCS clearly reveal the homogenous distribution of sulfur in the MIL-101 particles.

The XRD patterns of MIL-101(Cr) before and after sulfur infiltration are almost the same without any peaks implying the existence of sulfur crystals (Fig. 2(b)). Thus, the sulfur in the MIL-101(Cr) composite may be either amorphous or microcrystalline.^38^ In fact, by SEM and XRD results, it can be deduced that the sulfur in BPCS was in the form of nanoparticles that were uniformly dispersed inside the mesopores after the melting route, as seen subsequently from adsorption analysis.

N_2_ adsorption isotherms and the pore-size distribution (obtained by the DFT model) of the MIL-101(Cr), MIL-101(Cr)/S and BPCS composites are shown in Fig. 2(c). Sulfur infiltration dramatically decrease the BET surface area (from 3142 to 313 m^2^ g^−1^). It indirectly proves that sulfur was infiltrated into MIL-101(Cr), and preferentially filled rather than blocked the mesopores. The sulfur content measured with TGA (Fig. 1(b)), supports this argument. After biomolecule-doped PEDOT:PSS wrapping, the surface area was further decreased to less than 50 m^2^ g^−1^.

Thermogravimetric (TG) analyses were carried out to examine thermal stabilities for all the materials. Two main weight losses were observed for MIL-101(Cr). The first step of weight loss started at 30 °C and terminated at 150 °C. The corresponding weight loss should be attributed to the loss of water and solvent molecules accommodated in the cavities of MIL-101(Cr). The second step of the weight loss between 400 °C and 500 °C corresponded to the decomposition of the host frameworks, which is higher than the original report,^35^ and this is the result of using nitrogen as the carrier gas in the testing system, which is in agreement with the actual situation of carbonization. MIL-101/S and BPCS had no obvious weight loss before 150 °C, which can also reflect that most of the pores were covered by sulfur and biomolecule-doped PEDOT:PSS, which is consistent with the result obtained from BET. Comparing the thermodynamic stabilities of MIL-101(Cr)/S, BPCS have a slightly weight lost before 100 °C, which is due to the adsorbed H_2_O since the shell is very hydrophilic.^39^

Because MIL-101(Cr), BPCS and sulfur reduced their weights in the same temperature range (Fig. 1, 200–400 °C), it is impossible to measure the weight loss of the sulfur in BPCS by TG results. In order to estimate the weight percentage of sulfur (*W*_sulfur_), the weight changes were tested by subtractive method directly. The specific formula is shown in formulas (1) and (2). The quality of MIL-101 (*M*_MIL-101(Cr)_) remained unchanged before and after the impregnation of sulfur.

Therefore the content of sulfur in MIL-101(Cr)/S composite can be estimated through changes in the quality of samples (before (*M*_before_) and after (*M*_after_)). The concentration of PEDOT:PSS (f wt%) is about ∼0.94 wt% in BPCS and ∼1wt% in MIL-101/S@PEDOT:PSS, respectively.

1 *M*_MIL-101(Cr)_ = *M*_before_/3

2 *W*_sulfur_ = {(*M*_after_ − *M*_MIL-101(Cr)_)/*M*_after_} × {100/(100 + *f*)}

Through calculation, the sulfur content of BPCS and MIL-101/S@PEDOT:PSS is about 58.897% and 55.982%, respectively. Elemental analysis was adopted for further evaluate the sulfur content. The result was shown in Table 1, which is consistent with the result obtained from subtractive directly and significantly higher than that of some porous materials in the similar conditions.^16^

**Table 1:** Weight percentage of elements in different material

Sample	N [%]	C [%]	H [%]	S [%]
BPCS	0.57	13.43	0.652	57.884
MIL-101/S@PEDOT:PSS	0.54	23.31	2.184	54.066
MIL-101(Cr)	8.21	34.69	5.16	0.217

### Electrochemical measurements

**Fig. 4:**
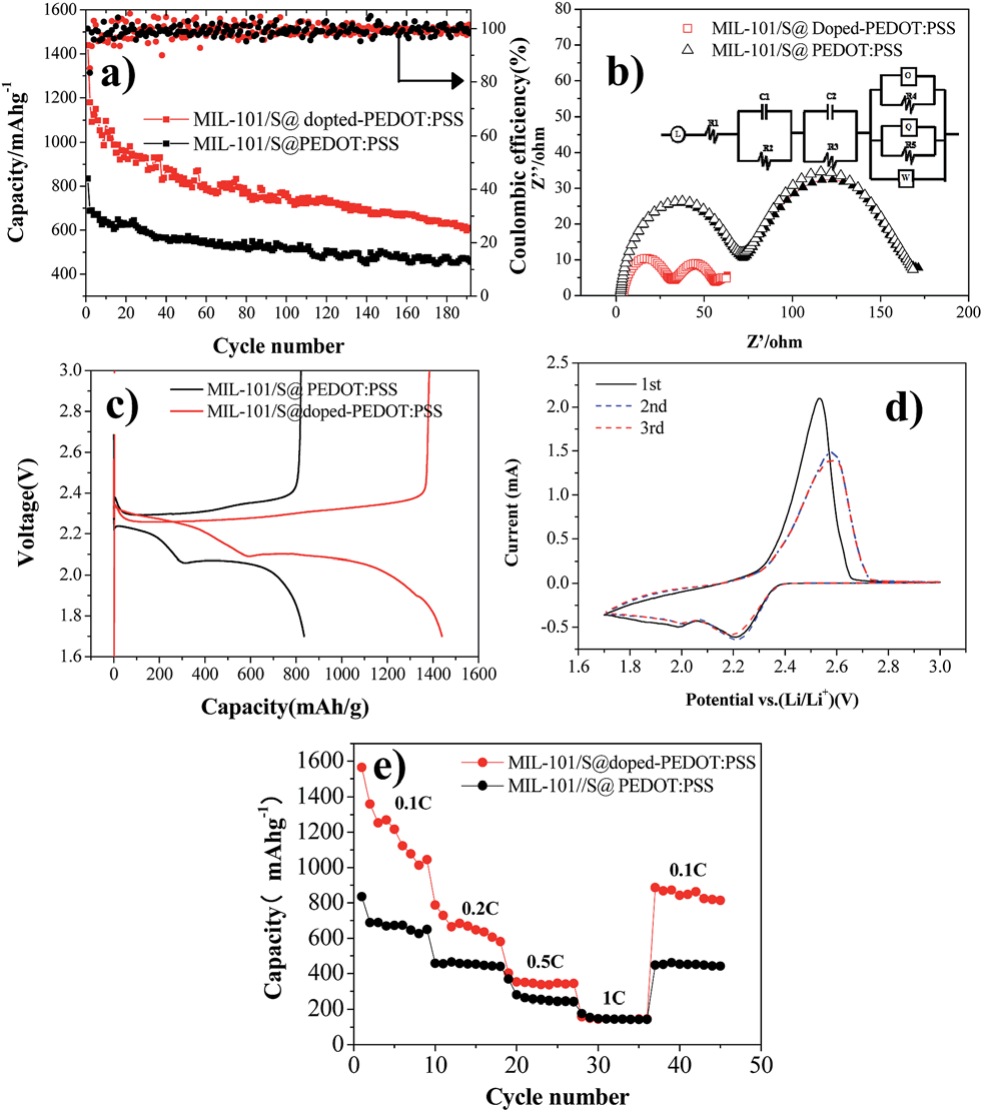
(a) Cycle performances of BPCS and MIL-101/S@PEDOT:PSS at 0.1C; (b) electrochemical impedance of BPCS and MIL-101/S@PEDOT:PSS electrodes and the equivalent circuit for the cell. Symbols denote experimental data, while the continuous lines represent the fitted data; (c) the first discharge–charge (0.1 C) curves of BPCS and MIL-101/S@PEDOT:PSS; (d) CV profiles of Li/S cells with BPCS composite cathodes (the potential sweep rate is 0.1 mV s^−1^); (e) rate performances of BPCS and MIL-101/S@PEDOT:PSS.

The electrochemical performance of BPCS and MIL-101/S@PEDOT:PSS composite was systematically measured by using cyclic voltammetry (CV), galvanostatic charge/discharge cycling tests and electrochemical impedance spectroscopy (EIS). Galvanostatic charge/discharge tests are shown in Fig. 4(a). At 0.1C, the APCNT–S composite shows much better cycling stability with 606.62 mA h g^−1^ reversible after 192 th cycles. In contrast, the discharge capacity of MIL-101/S@PEDOT:PSS cathode drops to 456.69 after 192th cycles.

It is obvious that the structure of BPCS helps increase the capacity retentions of the battery. The excellent electrochemical performance of BPCS electrode is derived from better contact of doped-PEDOT:PSS-encapsuled MIL-101/S nanoparticles with a conducting network, which results in easier transport of electrons across S nanoparticles and subsequently higher utilization of sulfur.

In addition, the structure of BPCS nanoparticles can tolerate 80% volume change during the charge/discharge process, which ensures good contact of MIL-101/S and doped-PEDOT:PSS shells and high discharge specific capacity.

Fig. 4(b) shows Nyquist plots of a lithium–sulfur pouch cell in electrochemical impedance spectroscopy (EIS) measurements for the BPCS and MIL-101/S@PEDOT:PSS before and after one cycle. The battery electrodes are usually porous, which leads to the impedance becoming inductive at high frequencies.^40,41^.Therefore, L refer to inductors associated with the cathode, *R*_1_ is the series resistance, corresponding to the sheet resistance of the contact resistance and the wire resistance. In this study, two semicircles were observed, which represent two layers just as they proposed.^42^

According to Fig. 4(b) this equivalent circuit represents an electrode surface, which has two layers on it, the one being inner and the other outer. This two-layer circuit design can be used for porous electrode surfaces, which have holes towards electrode conductive layer or one immobilization layer towards another.^42^ As you can see, there are two different semicircle diameter curves, and the important part of the electrode is the outer surface layer, which is the measurement layer, and *R*_2_ is the electron transfer resistance of the outer surface layer, *C*_1_ is the outer surface capacitance, while, *R*_3_ and *C*_2_ are two constants related to the inner surface (MIL-101/sulfur) that do not change. O represents the electron transfer resistance of sulfur enrichment zone between MIL-101 and doped-PEDOT:PSS. The high-frequency semicircle corresponds to the resistance (*R*_4_) of lithium ion diffusion through the SEI film.

The semicircle in the medium-frequency region is associated with the charge-transfer impedance (*R*_5_) and constant phase element (*Q*) of the electrode–electrolyte interface. Clearly, BPCS composite shows a much lower charge transfer resistance compared to the MIL-101/S@PEDOT:PSS composite. The straight line is assigned to the Warburg impedance (*Z*_w_) corresponding to the lithium-diffusion process.^43,44^ It is worth notice that the straight line of BPCS is much longer than MIL-101/S@PEDOT:PSS, which means that the lithium-diffusion process of MIL-101/S@doped-PEDOT:PSS is much more difficult. Fig. 4(c) presents the first discharge–charge voltage curve of BPCS and MIL-101/S@PEDOT:PSS at a rate of 0.1 C, which shows that the cathode of the BPCS composite delivers much higher discharge capacity of 1567.74 mA h g^−1^ with smaller polarization than the MIL-101/S@PEDOT:PSS cathode (835.21 mA h g^−1^). The upper branch (2.4–2.2 V) indicates the formation of polysulfide ions from sulfur located in large pores.^45^ The lower oblique branch (2.2–1.74 V) originates from the slow kinetics of lithium sulfide formation on the outer surface.^46^ CV curves of Li/S cell for first three cycles of BPCS composite cathode are shown in Fig. 4(d). Two broad reduction peaks centered at 2.2 V and 2.0 V were observed, corresponding to the two main stages of reduction reactions. The first peak is ascribed to the transformation of cyclooctasulfur(S_8_) into soluble long-chain lithium polysulfides and polysulfides to sulfur element, related to a fast kinetic reaction,^47–49^ while the other one originates from further decomposition of those polysulfides to insoluble Li_2_S_2_/Li_2_S, corresponding to the slow kinetics.^48,49^ One main peak associated with slow oxidation kinetics from lithium sulfides to lithium polysulfides and cyclooctasulfur dominates the subsequent electrochemical reaction.^48^ Compared with the first CV curves, the reduction peak at the second cycle shifts slightly towards higher voltage to 2.23 and 2.03 V (*vs.* Li/Li^+^). This could be ascribed to a weak polarization of the electrode after the first cycle.^9^ In subsequent scans, the overlapping cathodic and anodic peaks suggest superior cycle stability and highly reversible redox reactions. The typical redox of the material is all in agreement with their own galvanostatic charge–discharge curves (Fig. 4(c)).^50^ With the improvement of the electrical conductivity and the degree of coating, the doped-PEDOT:PSS serves as both the conductive network and containers to confine polysulfide species in cathode. Thus, the discharge capacity and rate performance of the BPCS composite are dramatically enhanced in comparison to the MIL-101/S@PEDOT:PSS composite.

The specific discharge capacities of the BPCS at 0.1 0.2, 0.3, 0.5 and 1C are 1439.71, 787.8, 402.2 and 158.1 mA h g^−1^, respectively, while the corresponding capacities of the MIL-101/S @PEDOT:PSS are only 835.21, 458.15, 368.85 and 175.9 mA h g^−1^. When the C rate was switched abruptly from 1 to 0.1C again (Fig. 4(e)), the original capacity of both composites were largely recovered, reflecting that the MIL-101/S@PEDOT:PSS cell and BPCS cell are robust and highly stable.^51^

## Experimental

### Preparation of MIL-101(Cr)crystals

The MIL-101(Cr) was synthesized by a following procedure reported previously. Cr(NO_3_)_3_·9H_2_O (99%+, from Aldrich), 1,4-benzene dicarboxylic acid (H_2_BDC, 99.0%+, from Acros), HF (48%, from Merck) were used as received without further purification.^35^ A typical synthesis involves a solution containing chromium(iii) nitrate Cr(NO_3_)_3_·9H_2_O (400 mg, 1 × 10^−3^ mol), 1 × 10^−3^ mol of fluorhydric acid, 1,4-benzene dicarboxylic acid H_2_BDC (164 mg, 1 × 10^−3^ mol) in 4.8 ml H_2_O (265 × 10^−3^ mol); the mixture is introduced in a hydrothermal bomb which is put during 8 h in an autoclave held at 220 °C. Then, the solution was filtered and the green colored precipitate was washed with DMF and ethanol (10 mL × 3 times) successively. Finally, the fine green colored crystals were obtained and dried in a vacuum oven at 150 °C overnight.

### Preparation of MIL-101(Cr)/S composite

The preparation of the MIL-101(Cr)/S composite was performed by following a melt-diffusion method. The MIL-101(Cr) crystals were first dried under vacuum at 150 °C overnight, and then a mixture of MIL-101(Cr) and elemental sulfur with a mass ratio of 1 : 2 were ground in ethanol for 0.5 h and heated at 155 °C for 12 h.

### Preparation of BPCS composite

Poly(3,4-ethylenedioxythiophene)/poly(styrene sulfonate) (PEDOT:PSS) solution was prepared by filtering commercially available solution (∼1 wt% solid content, Clevios PH1000) and adding 5 wt% dimethyl sulfoxide (DMSO), and 1 wt% Triton X-100 (biotechnology grade, MACKLIN, China) (final PEDOT:PSS concentration is ∼0.94 wt%).

Conductive-polymer solution and MIL-101(Cr)/S were mixed with a mass ratio of 1 : 10, and then the homogeneous mixture is milled in an agate mortar for about half an hour to reduce the particle sizes. The resulting powder was dried at 120 °C to remove water in the conductive-polymer solution, and the process is repeated several times till MIL-101(Cr)/S is fully wrapped in scale-like structures.

### Material characterization

Powder X-ray diffraction (XRD) patterns were recorded on a Bruker D8 Advance diffractometer using Cu-Kα radiation (40 kV and 200 mA). Data were collected from 2*θ* = 15° to 80° with a step of 0.02° and a scanning rate of 0.2° s^−1^. Nitrogen isotherms were measured at 77 K using an ASAP 2020 system (Micromeritics Co.). The samples were pretreated at 373 K and a pressure of less than 1.33 Pa for 1 h with further degassing at 473 K and a pressure of less than 26.7 Pa for 4 h. The specific surface area was calculated using the Brunauer–Emmett–Teller (BET) method based on adsorption data in the partial pressure (*P*/*P*_0_) range 0.10–0.20, and total pore volume was determined from the amount of nitrogen adsorbed at *P*/*P*_0_ = 0.99. Scanning electron microscopy (SEM) were carried out on a field emission SU-70 microscope. TEM images and elemental mapping images were obtained using a JEOL JEM2010 electron microscope. Thermogravimetric(TG) analysis was carried out on a TGA Q50 (TA Instruments) following the ASTM D3850-94 standard. The samples were heated from 25 to 500 °C under dry nitrogen at a constant heating rate of 10 °C min^−1^. All the samples were run in triplicate for thermal property measurements. Elemental analysis was carried out on an Elemental Vario MICRO CUBE (Germany).

### Cell fabrication and measurements

The electrochemical performance of these composites was tested using CR2025 coin-type cells fabricated in an Ar-filled glove box (O_2_ < 0.1 ppm; H_2_O < 0.1 ppm). The cathode slurry was prepared by mixing 80% of composite, 10% of Super-P and 10% of PVDF binder in 1-methyl-2-pyrrolidinone (NMP). The slurry was blade cast onto carbon-coated aluminium foil and dried at 60 °C for 12 h under vacuum. The weight load of S for MIL-101/S@PEDOT:PSS electrode and MIL-101/S@doped-PEDOT:PSS electrode is ∼0.8 mg cm^−2^ and ∼0.9 mg cm^−2^, respectively. The electrolyte was composed of 1 M bis(trifluoromethane) sulphonamide lithium salt and 0.1 M LiNO_3_ in a mixture of 1,3-dioxolane and 1,2-dimethoxyethane (1 : 1 by volume). The charge–discharge tests were conducted on NEWARE (Shenzhen, China) instruments (model 5V-10 mA) with voltage window of 1.5–2.6 V *versus* Li+/Li, and cycled at 0.1 C (1 C = 1670 mA g^−1^).

## Conclusions

In summary, a novel multi-core–shell with conductive network structured BPCS composite cathode for lithium–sulfur batteries has been synthesized. The initial discharge specific capacity of the BPCS composite cathode is 1439.71 mA h g^−1^, and remains a reversible capacity of 606.62 mA h g^−1^ after 192th cycles with a retention rate of 99.1%. Because of the special multi-core–shell of BPCS composite, the conductivity of the composite is obviously enhanced, and the diffusion of lithium polysulfides is effectively prevented.

Moreover, the mesoporous structure of the MIL-101(Cr) as a core structure has a higher surface area and pore volume, so it can be used to prepare high specific energy Li–S batteries. The BPCS composite is easily produced on a large scale, so it is a promising candidate for commercial Li–S batteries.

## Conflicts of interest

The authors declare that there is no conflict of interests regarding the publication of this article.

## Supplementary Material
